# Identification of Genes Differentially Expressed in *Myogenin* Knock-Down Bovine Muscle Satellite Cells during Differentiation through RNA Sequencing Analysis

**DOI:** 10.1371/journal.pone.0092447

**Published:** 2014-03-19

**Authors:** Eun Ju Lee, Adeel Malik, Smritee Pokharel, Sarafraz Ahmad, Bilal Ahmad Mir, Kyung Hyun Cho, Jihoe Kim, Joon Chan Kong, Dong-Mok Lee, Ki Yong Chung, Sang Hoon Kim, Inho Choi

**Affiliations:** 1 School of Biotechnology, Yeungnam University, Gyeongsan, Republic of Korea; 2 Bovine Genome Resources Bank, Yeungnam University, Gyeongsan, Republic of Korea; 3 Biomedical Manufacturing Technology Center, Korea Institute of Industrial Technology, Yeongcheon-si, Republic of Korea; 4 Hanwoo Experiment Station, National Institute of Animal Science, RDA, Pyeongchang, Republic of Korea; 5 Department of Biology, Kyung Hee University, Seoul, Republic of Korea; Ospedale Pediatrico Bambino Gesù, Italy

## Abstract

**Background:**

The expression of myogenic regulatory factors (MRFs) consisting of *MyoD*, *Myf5*, *myogenin* (*MyoG*) and *MRF4* characterizes various phases of skeletal muscle development including myoblast proliferation, cell-cycle exit, cell fusion and the maturation of myotubes to form myofibers. Although it is well known that the function of *MyoG* cannot be compensated for other MRFs, the molecular mechanism by which *MyoG* controls muscle cell differentiation is still unclear. Therefore, in this study, RNA-Seq technology was applied to profile changes in gene expression in response to *MyoG* knock-down (MyoG_kd_) in primary bovine muscle satellite cells (MSCs).

**Results:**

About 61–64% of the reads of over 42 million total reads were mapped to more than 13,000 genes in the reference bovine genome. RNA-Seq analysis identified 8,469 unique genes that were differentially expressed in MyoG_kd_. Among these genes, 230 were up-regulated and 224 were down-regulated by at least four-fold. DAVID Functional Annotation Cluster (FAC) and pathway analysis of all up- and down-regulated genes identified overrepresentation for cell cycle and division, DNA replication, mitosis, organelle lumen, nucleoplasm and cytosol, phosphate metabolic process, phosphoprotein phosphatase activity, cytoskeleton and cell morphogenesis, signifying the functional implication of these processes and pathways during skeletal muscle development. The RNA-Seq data was validated by real time RT-PCR analysis for eight out of ten genes as well as five marker genes investigated.

**Conclusions:**

This study is the first RNA-Seq based gene expression analysis of MyoG_kd_ undertaken in primary bovine MSCs. Computational analysis of the differentially expressed genes has identified the significance of genes such as *SAP30-like* (*SAP30L*), *Protein lyl-1* (*LYL1*), various matrix metalloproteinases, and several glycogenes in myogenesis. The results of the present study widen our knowledge of the molecular basis of skeletal muscle development and reveal the vital regulatory role of *MyoG* in retaining muscle cell differentiation.

## Introduction

Skeletal muscle formation is a multi-step process that requires proliferation of myocytes, expression of muscle-specific myogenic regulatory factors (MRFs) including *MyoD*, *Myf5*, *myogenin* (*MyoG*) and *MRF4* (or *Myf6*), cell cycle withdrawal, myotube formation by the fusion of mononucleated cells and maturation of myotubes into myofibers [1], [2], [3], [4], [5]. MRFs are basic helix-loop-helix (bHLH) transcription factors [6] that cooperate with several transcription factors of the *myocytes enhancer factor-2* (*MEF2*) family [7] to regulate myogenesis. bHLH proteins also heterodimerize with E-proteins [8], enabling binding to the E-Box consensus sequence (CANNTG) present in the regulatory regions of muscle specific genes [9], [10]. Among these MRFs, *MyoD* is highly expressed during the mid-G1 phase and between the S and M phases of the cell cycle, but absent during the G0 phase [11], whereas *Myf5* is highly expressed during the G0 phase and decreases during the G1 phase [12]. *MyoG* and *MRF4* (*Myf6*) are expressed upon differentiation of myoblasts into multinucleated myotubes [13], [14], [15].

*MyoG* is crucial during differentiation [11], as many studies have revealed that mice lacking *MyoG* continue to identify the muscle lineage through the formation of myoblasts [16], but show high perinatal mortality due to severe skeletal muscle deficiency caused by disruption of myoblast differentiation and muscle fiber formation [17], [18]. Additionally, *MyoG*/*MyoD* and *MyoG*/*Myf5* double knockout mice studies have shown that these mice specify the muscle lineage, but the formation of muscle fibers is disrupted, which is similar to *MyoG* knockout mice [19]. Furthermore, *MyoD* and *Myf5* are unable to compensate for the role of *MyoG* in differentiation [20], and mice that lack *MyoG* exhibit normal expression levels of *MyoD* and *Myf5* [17]. This is because *MyoG* acts downstream of *MyoD* and *Myf5* [16] in skeletal muscle differentiation. Knockout mice studies have also shown a relationship between different MRFs in which the absence of one will be compensated for by another [21], [22]. The only exception to this compensation effect of MRFs is *MyoG*, which plays a unique and non-redundant role during embryogenesis [19], whereas conditional knock-out resulted in reduced muscle mass in adults [23].

In this study, we conducted a comprehensive transcriptome analysis of primary bovine cells using MyoG_kd_ and compared the expression profiles with those of the wild type using an RNA-Seq technique. We also showed that MyoG_kd_ led to upregulation of genes involved in processes such as cell proliferation and DNA replication, whereas the genes involved in phosphate metabolic processes were down-regulated. Finally, potential involvement of various new genes in myogenesis was identified.

## Materials and Methods

### Bovine MSCs culture

Bovine muscle was collected from the hind leg skeletal muscle of 24–26 month old cattle with a body weight of 550–600 kg. The animals were handled according to a protocol approved by the Animal Care and Concern Committee of the National Institute of Animal Science, Korea. Briefly, the collected muscle was minced into fine pieces, and digested with trypsin-EDTA (GIBCO, CA, USA), and were centrifuged at 90×g for 3 min and the upper phase was passed through a 40-μm cell strainer. The filtrate was centrifuged at 2,500 rpm, pellet was collected, washed twice and cultured in Dulbecco's modified Eagle's medium (DMEM; HyClone Laboratories, UT, USA) supplemented with 10% fetal bovine serum (FBS, HyClone Laboratories) and 1% penicillin/streptomycin at 37°C under 5% CO_2_. The culture medium was changed every other day. To induce differentiation, cells were allowed to grow in DMEM without reducing serum (DMEM with 10% FBS and 1% P/S) for 10, 12, 14, 16, and 18 days. MSCs isolation and culture were conducted as previously described [24].

### *MyoG* shRNA construction and knock-down

Bovine *MyoG* shRNA was designed using nucleotide information obtained from NCBI (AB257560.1) and cloned with pRNAT-U6.2/Lenti vector (GeneScript, NJ, USA). Constructed *MyoG* shRNA or non-specific sequences (scrambled vector, MyoG_wd_) were transfected to generate viral particles in 293 FT cells. After two days of transfection, the supernatant containing viral particles was collected, transduced with lentiviral particles expressing shRNAs against bovine *MyoG* or scrambled vector in MSCs (Day 8), and selected with 50 μg/ml of G418 (CABIOCHEM, CA, USA). The selected cells were allowed to differentiate and were harvested at Day 21. The following oligonucleotide was used to generate *MyoG* shRNA: sense: 5′- GGATCCCGCGCAGACTCAAGCCGCCGGTGTTCAAGAGACACCTTCTTGAGTCTGCGCTTTTCCAACTCHGAG-3′.

### RNA extraction, library preparation and sequencing

MSCs were allowed to grow till day 10, and were transduced with either scrambled vector or *MyoG* shRNA. Cells were then allowed to grow for another 11 days, and were harvested with Trizol reagent (Invitrogen) according to the manufacturer's protocol. Total RNA was then extracted and stored in diethylpyrocarbonate-treated H_2_O at −80°C until used. The mRNA in total RNA was converted into a library of template molecules suitable for subsequent cluster generation using the reagents provided in the Illumina TruSeq RNA Sample Preparation Kit (Illumina, CA, USA) according to the manufacturer's instructions. Library construction and high-throughput sequencing were carried out using an Illumina HiSeq2000 sequencing system in which each sequencing cycle takes place in the presence of all four nucleotides, leading to higher precision than methods in which only a single nucleotide is present in the reaction mixture at one time. The cycle is repeated one base at a time, creating a string of images each indicating a single base extension at a specific cluster.

### Sequence quality check

The FASTQC [http://www.bioinformatics.babraham.ac.uk/projects/fastqc/] tool embedded in the web-based platform, Galaxy [25], [26], [27], was used to calculate quality control statistics describing raw sequence data from FASTQ files generated by the Illumina second generation sequencing technology (“Solexa”) [http://www.illumina.com/technology/solexa_technology.ilmn].

### Mapping of RNA-Seq reads transcript assembly

TopHat [28] was used to align RNA-Seq reads against UCSC *Bos taurus* reference genome (Btau_4.6.1/bosTau7) via Bowtie, which is a very high-throughput short read aligner [29]. Bowtie is different from other general-purpose alignment tools such as BLAST [30], and shows best performance when short reads are aligned to large genomes. Bowtie is extremely fast for short reads where several reads have at least one significantly valid alignment, the reads are of high-quality, and the number of alignments reported per read is nearly 1 [29]. These mapping results were then analyzed to identify splice junctions between exons. All default parameters were used to run TopHat except the mate inner distance, for which a value of 100 was selected in the case of paired reads. The advantage of a paired end run is that both reads contain long range positional information, allowing for highly precise alignment of reads.

The aligned reads were further analyzed by Cufflinks [31] using a multifasta file (bosTau7. fa) option that can improve the precision of transcript abundance approximation by bias detection and a correction algorithm. The relative abundance of transcripts was reported as fragments per kilobase of exon per million fragments mapped (FPKM). An additional cufflinks parameter for the initial estimation procedure was used so that the reads mapping to multiple locations in the genome were accurately weighted [31]. The nucleotide sequences obtained in this study have been submitted and will be available in NCBI Short Read Archive with accession number SRR1122446 as soon as it is released. Alternatively, the data can be obtained directly from the authors.

### Functional annotation cluster and pathway analysis

DAVID [http://david.abcc.ncifcrf.gov/home.jsp] functional annotation cluster analysis was performed on the list of up-regulated and down-regulated genes with a fold change of ≥4. Only those terms that reported a *p*-value of ≤0.05 and count number ≥5 genes were selected for analysis. The Gene Ontology (GO) terms of cellular component, molecular function and biological process in DAVID were employed to categorize enriched biological themes in up- and down-regulated gene lists. Pathway mapping was performed using the KEGG Automatic Annotation Server (KAAS) [32]. The nucleotide sequences of up- and down-regulated genes were uploaded to the KAAS web server as an input using single-directional best hit (SBH) method to assign orthologs. KAAS offers functional annotation of genes in a genome via a BLAST similarity searches against a manually curated set of ortholog groups in the KEGG GENES database. KAAS assigned a KEGG Orthology (KO) number to genes in the data sets, which were mapped to one of KEGG's reference pathways.

### Real time RT-PCR validation

One microgram of RNA in a reaction mixture with a total volume of 20 μl was primed with oligo (dT)_20_ primers (Bioneer, Daejeon, Korea) and then reverse transcribed at 42°C for 50 min and 72°C for 15 min. Subsequently, 2 μl of cDNA product and 10 pmoles of each gene-specific primer were used for PCR, using a 7500 real-time PCR system (Applied Biosystems, Foster City, CA, USA). A Power SYBRH Green PCR Master Mix (Applied Biosystems) was used as the fluorescence source. Primers were designed with the Primer 3 software (http://frodo.wi.mit.edu) using the sequence information listed at the National Center for Biotechnology Information. Detailed information describing the primer sequences is provided in **Table S1**.

### Immunocytochemistry

Cells grown in a covered glass-bottom dish were stained with Pax7 or MyoG antibody. Briefly, cells were rinsed with PBS (phosphate buffered saline), fixed in 4% formaldehyde, permeabilized by 0.2% TritonX-100, after which the signals were enhanced using an Image-iT FX signal enhancer (Invitrogen). The cells were then incubated with mouse primary Pax7 or MyoG antibody (1∶50, Santa Cruz Biotechnology, CA, USA) at 4°C in a humid environment overnight. Secondary antibody (Alexa Fluor 488 goat anti-mouse SFX kit; Molecular Probes, Eugene, OR, USA) was treated for 1 hr at room temperature followed by nuclear staining with 4′,6′-diamino-2-phenylindole (DAPI; Sigma-Aldrich, MO, USA). Pictures were taken using a fluorescent microscope equipped with a digital camera (Nikon, Tokyo, Japan).

### Western blot

Western blot was performed with the total protein isolated from cells. Briefly, cells washed with ice-cold PBS were lysed in RIPA lysis buffer containing protease inhibitor cocktail (Thermo Scientific, IL, USA). The protein was quantified by Bradford method using protein assay dye solution. Fifty microgram of protein was electrophoresed in 10% SDS-polyacrylamide gel after reducing at 90°C for 3 min with β-mercaptoethanol, and the protein was transferred to a PVDF membrane. Membrane was blocked and hybridized with MyoG (1∶1000) or β-actin antibody (1∶2000) (Santa Cruz Biotechnology) overnight at 4°C. Membrane washed in TBST was then incubated with horseradish peroxidase conjugated secondary antibody for an hour at room temperature. Finally, the membrane was developed using SuperSignal West Pico Chemiluminescent Substrate (Thermo Scientific).

### Giemsa staining

Cells were washed with PBS, fixed with PBS/methanol (v/v) for 2 min, and were incubated with 0.04% Giemsa G250 solution for 30 min. cells were rinsed with distilled water and pictures were taken using a light microscope equipped with a digital camera (Nikon).

### Statistical analysis

The normalized expression means were compared using Tukey's Studentized Range (HSD) to identify significant differences in gene expression. A nominal *p*-value of less than 0.05 was considered to be statistically significant. Real time RT-PCR data were analyzed by one-way ANOVA using PROC GLM in SAS package ver. 9.0 (SAS Institute, Cary, NC, USA).

## Results

### *MyoG* gene knock-down

**Figure 1:**
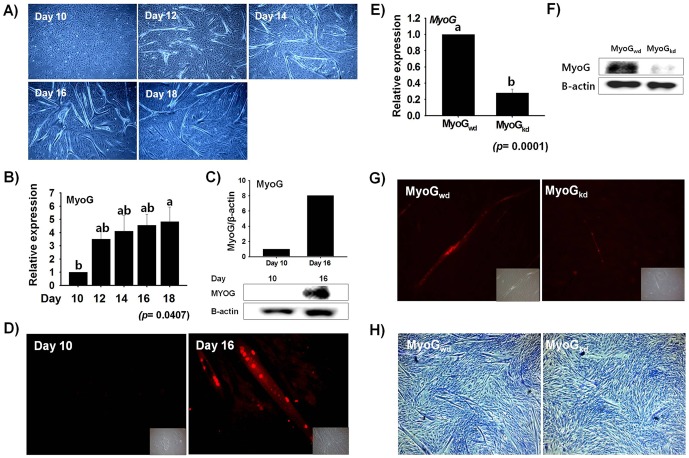
Myogenesis is associated with increased *MyoG* expression. **A**) MSCs proliferation and differentiation in DMEM/10% FBS media. Myotube formation was observed from day 12, reaching a maximum at day 16. **B**) *MyoG* expression from RNA extracted and analyzed by real time RT-PCR. *MyoG* expression gradually increased with time. **C**) MyoG protein expression at Day 10 and Day 16 was determined by Western blot analysis and the protein intensity was measured by using Image J program. MyoG protein in Day 16 cells was significantly higher than in Day 10 cells. **D**) Immunocytochemistry analysis of Day 10 and Day 16 cells stained with MyoG antibody. Day 16 showed higher MyoG nuclear expression as compared with Day 10 proliferating cells. **E)& F)** *MyoG* mRNA and protein expression of MSCs transfected with MyoG shRNA. *MyoG* expression was decreased in both RNA and protein level in MyoG_kd_ cells relative to MyoG_wd_ cells. **G**) Immunostaining of MyoG_wd_ or MyoG_kd_ cells with MyoG antibody. A significant decrease in nuclear MyoG protein expression was observed in MyoG_kd_ cells. **H**) Giemsa staining performed in MyoG_wd_ and MyoG_kd_ cells. Myotube formation was decreased in MyoG_kd_ cells relative to MyoG_wd_ cells. Day 10 and MyoG_wd_ represents control, respectively (mean ±S.D., n = 3). *p*-value indicates the statistical significance of the data and different letters (a and b) in graph show significant differences among groups.

MSCs isolated and cultured from bovine leg muscle were stained with Pax7 to determine the purity of cells. The isolated cells showed approximately 85% Pax7 positive cells (**Figure S1**). The *in vitro* cultured bovine primary cells began to differentiate without serum deprivation. The initial myotubes became visible at Day 12, and the number of myotubes increased with time (**Figure 1A**). The expression level of *MyoG*, which is known to play a role in myogenic differentiation [33], was determined by real-time RT-PCR at different time points of primary bovine cells differentiation. *MyoG* was expressed throughout differentiation; however, there was a steep increase in its expression level at Day 12, which gradually increased until Day 18 (**Figure 1B**). Similarly, Western blot analysis revealed MyoG protein expression on Day 10 and Day 16 with higher levels occurring on Day 16 (**Figure 1C**). This expression profile of *MyoG* during differentiation is in accordance with those of previous studies [34], [35]. Moreover, the cells were authenticated to be in the state of myotube formation by inspecting the nuclear expression of MyoG protein at two different time points (Day 10 and Day 16) during cell differentiation. MyoG protein expression was observed at Day 10 and 16. Day 16 showed higher MyoG nuclear expression as compared with Day 10 proliferating cells (**Figure 1D**). To identify the genes differentially expressed as a consequence of *MyoG* knock-down, MSCs were transduced with shRNA specific for *MyoG*. RNA analysis following transduction revealed the specific decline of mRNA for shRNA induced MyoG_kd_ as compared to its wild type counterpart (MyoG_wd_) (**Figure 1E**). Similarly, MyoG_kd_ was confirmed at the protein level by Western blot analysis (**Figure 1F**). shRNA transduction against *MyoG* prohibited the nuclear expression of MyoG protein and the development of myotubes (**Figure 1G and 1H**).

### Expression of myogenic marker genes

**Figure 2:**
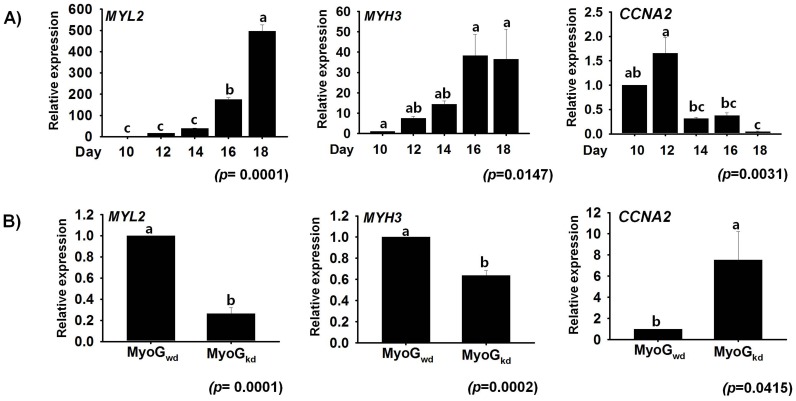
Effect of MyoG_kd_ on *MYL2*, *MYH3* and *CCNA2* genes. **A**) Time course study of mRNA expression of *MYL2*, *MYH3* and *CCNA2* during MSCs differentiation. Cells cultured in differentiation media showed gradual increase in *MYL2* and *MYH3* expression until Day 18, but transient increase of *CCNA2* at day 12 decreased at later stage of myogenesis. **B**) Evaluation of *MYL2*, *MYH3*, and *CCNA2* gene expression by real time RT-PCR in MyoG_kd_ cells. Decreased *MYL2* and *MYH3* gene expression and increased *CCNA2* expression was observed in MyoG_kd_ cells relative to MyoG_kd_ cells. Day 10 and MyoG_wd_ represents control, respectively (mean ±S.D., n = 3). *p*-value indicates the statistical significance of the data and different letters (a, b and c) in graph show significant differences among groups.

To confirm that primary bovine cells were undergoing differentiation, we verified the expression of *myosin regulatory light chain 2* (*MYL2*) and *myosin heavy chain 3* (*MYH3*), which have previously been shown to be expressed during myogenesis. Both *MYL2* and *MYH3*, which are marker genes [24], exhibited a gradual increase in expression rates during myogenesis, whereas *cyclin A2* (*CCNA2*), which is involved in the cell cycle [36], showed moderate and decreased expression levels (**Figure 2A**). The opposite trend was observed for *MYL2* and *MYH3*, with decreased mRNA expression, while the expression of *CCNA2* was significantly elevated as a result of MyoG_kd_ (**Figure 2B**). These results are in agreement with those of our previous study [37], [38], as well as those of other investigations of mouse and human skeletal muscle differentiation [4], [39].

### High-throughput sequencing

**Figure 3:**
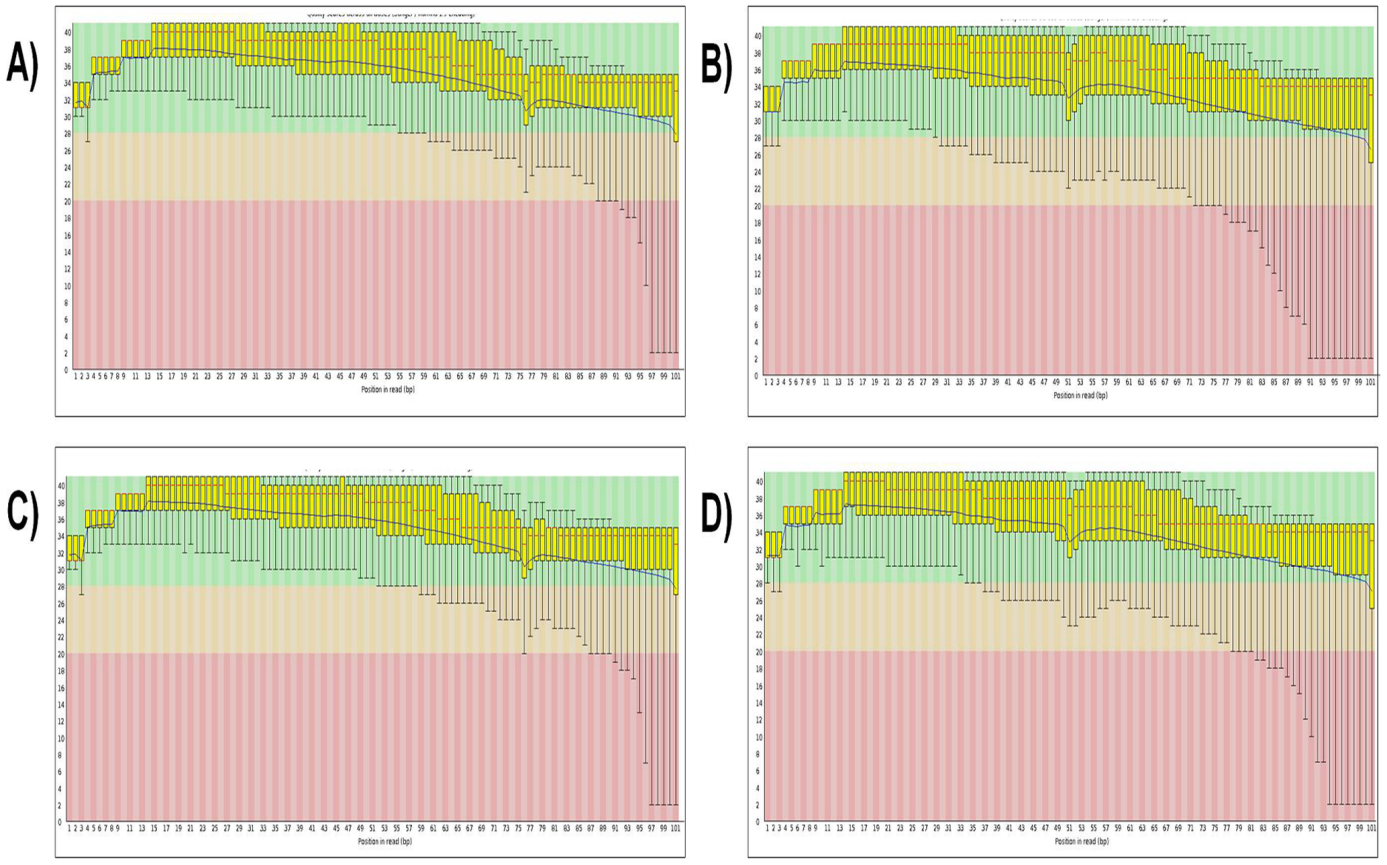
Per base sequence quality of MyoG_wd_ and MyoG_kd_. Quality scores of **A) & B)** MyoG_wd_, and **C) & D)** MyoG_kd_. The y-axis on the graph shows the quality scores with higher scores indicating better base calls. The background of the graph separates the y axis into high-quality calls (green), calls of reasonable quality (orange), and calls of poor quality (red). In each of these findings, the red line is the median value, the yellow box corresponds to the inter-quartile range (25–75%), the upper and lower whiskers represent the 10% and 90% points, respectively, and the blue line signifies the mean quality.

High-throughput RNA-Seq was applied to investigate the gene expression profiles of MyoG_wd_ and MyoG_kd_ samples. The total numbers of RNA-Seq reads (101 base pairs in length) generated in this study were about 42 million for MyoG_wd_ and 46 million for MyoG_kd_. About 63.73% and 61.66% of the MyoG_wd_ and MyoG_kd_ reads, with at least one reported alignment, were mapped to the reference genome (**Table 1**). Post-run quality analysis of the RNA-Seq data was carried out using the FASTQC [http://www.bioinformatics.babraham.ac.uk/projects/fastqc/] tool in Galaxy [25], [26], [27]. The per base sequence quality report is one of the most useful FASTQC reports, which helps in deciding whether sequence trimming is required before alignment. **Figure 3A–D** summarizes the range of quality standards across all bases at every point in the FastQ file. For each position a boxwhisker type plot is drawn. In general, the quality of calls will degrade as the run advances, therefore, it is prevalent to see base calls falling into the orange area towards the end of a read. The quality scores across all bases were determined by the Sanger/Illumina 1.9 encoding method. These figures represent good quality calls scattered across the green background of the plots. A warning will be pointed out if the lower quartile for any base is less than 10, or if the median for any base is less than 25, whereas a failure will be issued if the lower quartile for any base is less than 5 or if the median for any base is less than 20.

**Table 1:** Number of single replicate RNA-Seq reads across MyoG_wd_ and MyoG_kd_ samples.

Sample	Total read pairs^a^	Processed reads^b^	Mapped reads^c^
**MyoG_wd_**	42,936,654	42,910,546	27,346,293 (63.73%)
**MyoG_kd_**	46,349,131	46,324,224	28,565,046 (61.66%)

Foot note: a. Total read count b. Reads used in TopHat process c. Reads with at least one reported alignment.

### Differentially expressed genes

Following mapping of the sequencing reads to the reference genome with TopHat [28], transcripts were assembled and their relative expression levels were computed with Cufflinks [31] in FPKM. A total of 13,703 unique genes were detected and further filtered to remove possible noise from the data by excluding the genes with FPKM values equal to zero from the analysis. As a result, 9,337 and 12,835 genes were identified from MyoG_wd_ and MyoG_kd_ samples respectively, which shared 8,469 genes in common (**Table 2**). These 8,469 genes were then used to calculate the fold change, which was defined as the ratio of MyoG_kd_ FPKM to MyoG_wd_ FPKM. In this study, the total fold change of ≥4 was considered to classify the differentially expressed genes. Based on this definition, there are 230 up-regulated and 224 down-regulated genes in MyoG_kd_ over the MyoG_wd_ sample (**Table S2**). We found that *SAP30-like* (*SAP30L*) was the most up-regulated gene in MyoG_kd_ by 126-fold (log_2_ fold change  = 6.98) and encodes a protein that plays a potential role in the histone deacetylase complex, similar to Sin3 associated protein 30 (SAP30) [40].

**Table 2:** Gene expression summary.

Sample	MyoG_wd_	MyoG_kd_
Total No. of genes	9,337	12,835
Common in MyoG_wd_/MyoG_kd_		8,469
Up-regulated (≥4 fold)		230
Down-regulated (≥4 fold)		224

*SAP30* was identified as one of the transcriptional regulators in C2C12 differentiation [41]. *Ribosomal protein L23a* (*RPL23A*), *zinc finger protein 322A (ZNF322A)*, *solute carrier family 16 member 3* (*SLC16A3*), *tubulin, alpha 1c* (*TUBA1C*), *sulfotransferase family, cytosolic, 2B, member 1* (*SULT2B1*), *metallothionein 2A* (*MT2A*), *matrix metallopeptidase 9* (*MMP9*), *secreted frizzled-related protein 1* (*SFRP1*) and *myelin protein* (*MBP*) were among the ten most up-regulated genes in MyoG_kd_. *MT2A*, a member of cysteine-rich and metal binding intracellular proteins [42] that has been linked with cell proliferation [43], was up-regulated by 17.77-fold. In addition to *MT2A*, the other two metallothioneins (*MT1A* and *MT1E*) present in the list of differentially expressed genes also showed up-regulation by more than four-fold. Another group of highly up-regulated genes belonged to a class of matrix *metalloproteinases* (*MMPs*). A total of 13 *MMPs* were identified in this study, almost all of which showed a high fold change. *MMP1*, *MMP3*, *MMP9* (one of the ten most up-regulated genes) and *MMP13* were up-regulated by more than four-fold, whereas *MMP2* and *MMP12* were up-regulated by more than two-fold. Similarly, *MMP15*, *MMP19*, *MMP20*, *MMP23B* and *MMP27* were up-regulated by more than one-fold. Only one of the *MMPs*, *MMP16*, was down-regulated. Increased expression of *MMPs* (particularly *MMP9*) in skeletal muscles is well known [44]; therefore, our analysis is consistent with the results of previous studies. These *MMPs* enable release of the active *hepatocyte growth factor* (*HGF*), which stimulates proliferation while inhibiting differentiation, from extracellular matrix (ECM) [45]. The other genes that showed a greater than four-fold increase in expression include a transcription repressor, *musculin* also known as *MyoR* (myogenic repressor), which is known to block myogenesis and the activation of E-box dependent muscle genes [46].

*Protein lyl-1* (*LYL1*), also known as *lymphoblastic leukemia-derived sequence 1*, was found to be one of the ten most down-regulated genes. *LYL1* consists of a basic helix-loop-helix (bHLH) domain (**Figure S2**), which is similar to genes involved in mammalian myogenesis (*MyoD*, *MyoG*, *Myf5*, and *herculin*) [47]. *LYL1* is an essential gene required for the development of adult hematopoietic stem cells [48]. The other ten most down-regulated genes include *sodium channel protein type 1 subunit alpha* (*SCN1A*), *ribosomal protein S15a* (*RPS15A*), *syncoilin* (*SYNC*), *tubulin alpha-1D* (*TUBA1D*), *family with sequence similarity 65, member B* (*FAM65B*), *agouti-signaling protein* (*ASIP*), *tocopherol (alpha) transfer protein-like* (*TTPAL*), *ryanodine receptor 1* (*RYR1*), and an uncharacterized protein (LOC100847946). *Myocyte enhancer factor 2C* (*MEF2C*) is a member of the *MEF2* family of transcription factors that was down-regulated by two-fold, whereas another member of the *MEF2* family, *MEF2A*, was down-regulated by about 1.5-fold. *MEF2* transcription factors play prominent roles in skeletal muscle differentiation [49], [50], and four *MEF2* isoforms (*MEF2A*, *MEF2B*, *MEF2C* and *MEF2D*) have been identified to date [51], [52]. It is well known that increased expression of *MEF2C* occurs during myoblast differentiation [53], [54], [55]. In comparison with the up-regulated genes, down-regulated genes consisted of those that play crucial roles in phosphate metabolic processes. There is a significant amount of evidence that the processes related to phosphorylation and dephosphorylation of tyrosine are important regulatory components during the progression of myogenesis [56], [57], [58], [59].

### Functional annotation cluster and pathway analysis

To categorize biological processes that are overrepresented in MyoG_wd_ and MyoG_kd_ cells, we classified all known differentially expressed genes (fold change ≥4) using the Functional Annotation Cluster (FAC) tool available in the Database for Annotation, Visualization and Integrated Discovery (DAVID) [http://david.abcc.ncifcrf.gov/home.jsp]. DAVID FAC analysis of 230 up-regulated genes (fold change ≥4) generated a total of 65 functional clusters using default parameters. The GO terms “Biological Process”, “Cellular Component” and “Molecular Function” were used for annotations. Genes with a variety of GO terms from the resulting functional clusters having statistically significant *p*-values are listed in **Table 3A**. Similarly, the GO functional annotation chart reported 50 chart records that were further filtered to 22 records by selecting only those terms having *p*-values ≤0.05 and number of genes in each term ≥5 (**Table S3A**). From these tables, it can be seen that the GO terms are enriched in genes with functions necessary for actively proliferating cells such as cell division, DNA replication, cell cycle function and mitosis. The other major processes that exhibit higher levels of gene expression as a consequence of MyoG_kd_ include GO terms related to organelle lumen, nucleoplasm and cytosol. These data suggest that the proliferating processes are the major processes up-regulated by MyoG_kd_, and that their overrepresentation may be due to the de-differentiation of muscle cells. The down-regulation of *MyoG* in terminally differentiated mouse C2C12 myotubes was recently shown to stimulate cellular cleavage into mononucleated cells and promote cell cycle re-entry [11]. This phenomenon of dedifferentiation of myotubes into proliferating mononucleated cells is well known [60], [61], [62].

**Table 3:** Significantly enriched gene ontology terms detected by FAC in A) up-regulated genes, and B) down-regulated genes.

	S. No.	GO Term (Fold enrichment)	No. of Genes	P-Value
**A) Up-regulated**	**1.**	GO:0005654∼nucleoplasm (1.92)	22	0.0047
	**2.**	GO:0031981∼nuclear lumen (1.54)	29	0.0189
	**3.**	GO:0031974∼membrane-enclosed lumen (1.45)	35	0.0203
	**4.**	GO:0043233∼organelle lumen (1.44)	35	0.0254
	**5.**	GO:0070013∼intracellular organelle lumen (1.43)	33	0.0306
	**6.**	GO:0005615∼extracellular space (1.91)	17	0.0157
	**7.**	GO:0044421∼extracellular region part (1.68)	21	0.0227
	**8.**	GO:0004222∼metalloendopeptidase activity (3.85)	5	0.0404
	**9.**	GO:0043933∼macromolecular complex subunit organization (1.76)	16	0.037
	**10.**	GO:0000280∼nuclear division (2.84)	8	0.0225
	**11.**	GO:0007067∼Mitosis (2.84)	8	0.0225
	**12.**	GO:0000087∼M phase of mitotic cell cycle (2.79)	8	0.0245
	**13.**	GO:0048285∼organelle fission (2.73)	8	0.0272
	**14.**	GO:0051301∼cell division (2.39)	9	0.0349
	**15.**	GO:0007049∼cell cycle (1.71)	17	0.0384
	**16.**	GO:0005819∼Spindle (3.14)	6	0.0418
	**17.**	GO:0005875∼microtubule associated complex (3.77)	5	0.043
	**18.**	GO:0000278∼mitotic cell cycle (2.11)	10	0.0469
	**19.**	GO:0007346∼regulation of mitotic cell cycle (3.09)	6	0.0447
**B) Down-regulated**	**1.**	GO:0004721∼phosphoprotein phosphatase activity (3.51)	7	0.0146
	**2.**	GO:0006470∼protein amino acid dephosphorylation (3.72)	6	0.0224
	**3.**	GO:0016311∼dephosphorylation (3.21)	6	0.0387
	**4.**	GO:0043292∼contractile fiber (4.06)	6	0.0159
	**5.**	GO:0030017∼sarcomere (4.18)	5	0.0312
	**6.**	GO:0030016∼myofibril (3.69)	5	0.046
	**7.**	GO:0044449∼contractile fiber part (3.63)	5	0.0486
	**8.**	GO:0000904∼cell morphogenesis involved in differentiation (3.38)	10	0.0028
	**9.**	GO:0000902∼cell morphogenesis (2.55)	11	0.0111
	**10.**	GO:0032989∼cellular component morphogenesis (2.29)	11	0.022
	**11.**	GO:0031175∼neuron projection development (2.58)	8	0.0355
	**12.**	GO:0048666∼neuron development (2.19)	9	0.0529
	**13.**	GO:0016049∼cell growth (8.11)	6	0.0008
	**14.**	GO:0040007∼growth (3.61)	8	0.0067
	**15.**	GO:0008361∼regulation of cell size (3.20)	8	0.0126
	**16.**	GO:0032535∼regulation of cellular component size (2.74)	9	0.017
	**17.**	GO:0031090∼organelle membrane (1.64)	22	0.0239
	**18.**	GO:0031966∼mitochondrial membrane (2.08)	10	0.0506
	**19.**	GO:0005874∼microtubule (2.69)	9	0.0186
	**20.**	GO:0005856∼cytoskeleton (1.48)	25	0.0445
	**21.**	GO:0006796∼ phosphate metabolic process (1.70)	20	0.0251
	**22.**	GO:0006793∼phosphorus metabolic process (1.70)	20	0.0251
	**23.**	GO:0030424∼axon (3.09)	6	0.0443

Functional analysis of 224 down-regulated genes resulted in 69 clusters, and the statistically significant (*p*-value ≤0.05) GO terms having at least five members in each enriched term are listed in **Table 3B**. The GO functional annotation chart reported 59 records, 21 of which were selected on the basis of a *p*-value ≤0.05 and number of genes in each term ≥5 (**Table S3B**). The processes that were significantly down-regulated by MyoG_kd_ as shown by functional analysis include phosphate metabolic processes, dephosphorylation, phosphoprotein phosphatase activity and protein amino acid dephosphorylation. Additionally, processes related to the cell shape such as cytoskeleton and cell morphogenesis (**Table 3B** **& Table S3B**) were also down-regulated. In addition to DAVID FAC, by performing KEGG pathway analysis of 455 up- and down-regulated genes, we were able to assign 281 unique KEGG orthologs to these differentially expressed query genes. The majority of the differentially expressed genes were found to be associated with important biological processes, many being classified in signaling pathway or being involved in adhesion and cytoskeleton related functions (**Figure S3A–S3E**).

### Glycogenes expression

To further explore the role of glycogenes in myogenesis, all 230 up- and 224 down-regulated genes were manually verified in the UniProt database [63] to check whether they represent a glycogene or not. If a gene encoded a protein and represented glycosyltransferases, glycosidases, lectins, sulfotransferases or proteins involved in carbohydrate metabolism or transport [5], it was labeled as a glycogene. In this way, we identified 59 (∼25%) up- and 52 (∼23%) down-regulated glycogenes out of 230 and 224 differentially expressed gene sets. Some of the glycogenes that demonstrated four-fold increase in expression rates included *SCNN1A*, *SFRP1* and *transmembrane protein 217* (*TMEM217*). Additionally, various glycogenes such as *matrix metalloproteinases* (*MMP1*, *MMP13*, *MMP3* and *MMP9*) and genes belonging to the solute carrier family (*SLC26A8*, *SLC2A6*, *SLC37A2* and *SLC46A3*) exhibited more than four-fold up-regulation as a consequence of MyoG_kd_. Similarly, the glycogenes that showed four-fold decrease included *SCN1A*, *ASIP* and *protein wnt-11* (*WNT11*). The list of top ten up- and down-regulated genes consisted of at least two glycogenes.

### Validation of RNA sequencing data

**Figure 4:**
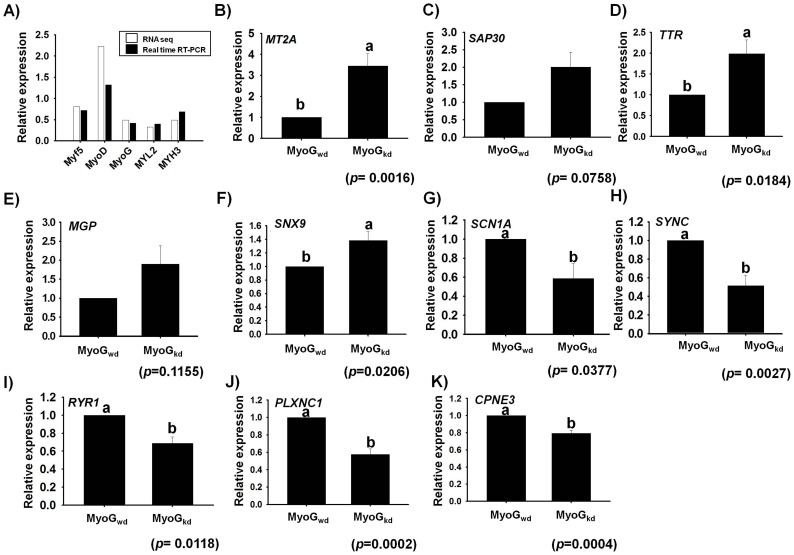
Real time RT-PCR validation of muscle specific and differentially expressed genes on MyoG_kd_. **A**) Fold changes of *Myf5*, *MyoD*, *MyoG*, *MYL2*, and *MYH3* genes determined by RNA sequencing were compared with real-time RT PCR results. **B–K**) RT-PCR validation of mRNA expression for five randomly selected genes confirmed the increased expression of *MT2A SAP30*, *TTR*, *MGP*, and *SNX9* genes and decreased expression of *SCN1A*, *SYNC*, *RYR1, PLXNC1*, and *CPNE3* genes by MyoG_kd_. MyoG_wd_ represents control, respectively (mean ±S.D., n = 3). *p*-value indicates the statistical significance of the data and different letters (a and b) in graph show significant differences among groups.

To validate the RNA-Seq results, we performed real-time RT-PCR to determine the expression levels of marker genes (*Myf5*, *MyoD*, *MyoG*, *MYL2* and *MYH3*) involved in myogenesis and then compared their expression with RNA-Seq data in MyoG_kd_ samples. The RT-PCR results were well correlated with the RNA-Seq expression data for the five marker genes investigated (**Figure 4A**). Specifically, RT-PCR analysis of *Myf5*, *MyoD*, *MyoG*, *MYL2* and *MYH3* mRNA levels revealed fold changes of 0.72, 1.32, 0.42, 0.35 and 0.67, respectively (approximately >2-fold decrease) in MyoG_kd_ relative to MyoG_wd_ cells. These results compliment favorably well with our RNA-Seq data showing fold changes of 0.8 (*Myf5*), 2.23 (*MyoD*), 0.49 (*MyoG*), 0.33 (*MYL2*) and 0.49 (*MYH3*), which also corresponded to a two-fold decline in the expression of these marker genes. As an additional confirmation of the expression data, ten genes were randomly selected for RT-PCR analysis, five of them representing the ten most-up and down-regulated genes. The results revealed that the fold-change profiles measured by RNA-Seq and RT-PCR were concordant for all ten genes. However, RT-PCR analysis showed statistically significant expression of only eight out of these additional ten genes (**Figure 4B–K**). Among the RNA-Seq data, SAP30, *MT2A, sorting nexin 9* (*SNX9*), *transthyretin* (*TTR*), *and matrix gla protein* (*MGP*), were up-regulated 126.47-fold, 17.77-fold, 5.75-fold, 2.5-fold, and 2.1-fold, respectively. RT-PCR also indicated an approximately four-fold increase in expression for SAP30, MT2A whereas TTR and MGP showed about 2 fold increase in their expression. However, RT-PCR results of SNX9 exhibited about 1.4-fold increase. Similarly, *SCN1A*, *SYNC*, *RYR1, plexin 1* (*PLXNC1*), and *copine III* (*CPNE3*) were down-regulated by 0.01-fold, 0.02-fold, 0.08-fold, 0.13-fold, and 0.17-fold (>4-fold), respectively. However, the RT-PCR data indicated an approximately two-fold decrease in their expression levels.

## Discussion

The current study offers the first thorough insight into the transcriptome analysis of primary bovine MSCs with MYOG_kd_ using RNA-Seq technology. The number of total reads that map to the reference genome met the high quality criterion of the RNA-seq technology [64]. The most practical justification for reads not mapping uniquely to the reference genome could be due to the sequencing errors or polymorphisms, reads that come from repetitive sequences, and reads from exon-exon junctions [65].

Several genome wide high-throughput studies have been applied to investigate the functional role of various genes during myogenesis [4], [39], [66], [67]. Recently, a microarray based study of *MyoG* has shown its role in mediating cell cycle exit in the absence of *p38α* and recognized an important function of *p38α* in cell fusion through the up-regulation of *CD53* [68]. Another DNA microarray based study identified approximately 193 additional transcriptional regulators with varying expression levels during myogenesis [41]. DNA microarray has also been used to observe global changes in C2C12 cells transcriptome stimulated by exogenous *myostatin* (also known as *GDF8*) treatment, as well as to identify a network of genes involved in the inhibitory effects on differentiation [69]. In addition to microarray based studies, the RNA-Seq technique has been applied using C2C12 mouse myoblast cell lines to detect 13,692 known transcripts and 3,724 unannotated transcripts [29]. These sequencing or array-based methods have been shown to improve our understanding of myogenesis by revealing a broad range of target genes of myogenic transcription factors, novel myogenic factors and the characteristics of myoblasts and myotubes, which are difficult to identify by traditional approaches.

However, almost all of the aforementioned studies have used C2C12 mouse cell lines. We recently used primary bovine cells of high purity [70] to identify genes differentially expressed during differentiation and transdifferentiation of MSCs and differentiation of preadipocytes [37], [70]. MSCs are stem cells that reside between the sarcolemma and the basal lamina of adult skeletal muscle [71]. Since the serum derived from bovine species is an essential component of the *in vitro* cell culture system, there would be a great advantage of using bovine primary MSCs that closely mimic the *in vivo* situation during myogenesis [72]. Indeed, such studies might enable enhancement of muscle fiber characteristics, leading to improved meat quality. *MyoG* is specifically responsible for muscle fiber characteristics and closely associated with meat quality by affecting muscle development [73], [74].

### Key processes altered during MyoG_kd_

#### Genes involved in cell cycle regulation and DNA replication

MyoG_kd_ caused up-regulation of a large number of genes involved in functions related to cell proliferation, such as DNA replication, the cell cycle and mitosis (**Table 3A** **& Table S3A**). **Figure 2B** verified the computational results showing that *CCNA2* expression increased by more than ten-fold in response to MyoG_kd_ based on real time RT-PCR. Cell cycle related genes in these groups consist of several cell division homologue genes including *cell division cycle 45* (*CDC45*), *cell division cycle 20* (*CDC20*) and *cell division cycle 6* (*CDC6*), each showing more than a four-fold change. Promoter studies in quiescent myoblasts have shown that *MyoD* activates the expression of *CDC6* and *minichromosome maintenance complex component 2* (*MCM2*) genes, which prepare chromatin for DNA replication and as a result progression of the cell through the S-phase. Additionally, several other key cell division cycle-associated proteins including *cell division cycle associated 2* (*CDCA2*), *cell division cycle associated 3* (*CDCA3*), *cell division cycle associated 7* (*CDCA7*) and *cell division cycle 8* (*CDC8*) were up-regulated in response to MyoG_kd_. *MyoG* plays a critical role in mediating terminal differentiation through cell cycle exit, and the activation of several cell cycle genes as a consequence of *MyoG* down-regulation is well known [68]. Among the transcription factors, *E2F transcription factor 1* (*E2F1*), which is known to play important roles in regulating cell proliferation [75], was up-regulated by about seven-fold. Previous studies have demonstrated that the expression of *MyoG* is strongly correlated with miRNA (miR-20a) expression, which in turn controls cell cycle exit by targeting E2F transcription factors [68], [76], [77], [78]. Two high mobility group box genes (*HMG20B* and *HMGA1*) that play a role in the regulation of DNA-dependent processes (transcription, replication, and DNA repair) involved in altering the conformation of chromatin also belong to this group of up-regulated biological processes [79]. Moreover, expression of MCM proteins (putative replicative helicase) such as *MCM5* is necessary for DNA replication [80], [81] and essential for DNA replication fork progression [82], [83].

#### Processes related to organelle lumen, nucleoplasm and cytosol

DAVID functional analysis identified 59 unique genes up-regulated by more than four-fold representing the GO terms organelle lumen, nucleoplasm and cytosol (**Table 3A** **and Table S3A**). These genes included transcription factor *E2F1*, *endoplasmic reticulum resident protein 44* (*ERP44*), *gamma-enolase* (*ENO2*) and *vascular endothelial growth factor-D* (*VEGF-D*), which are involved in a wide variety of biological processes. The proteins belonging to the E2F family of transcription factors play a significant role in controlling cell proliferation. For example, *E2F1* is considered the key target of pRB and is regulated by pRB throughout cell proliferation [84]. *ERP44*, a *thioredoxin* (*TRX*) family protein known to be involved in oxidative protein folding, directly regulates or inhibits the channel activity of *inositol 1,4,5-trisphosphate receptors* (*IP_3_Rs*). *ERP44* exclusively interacts with the L3V domain of *IP_3_R1*, and this binding is dependent on pH, Ca^2+^ concentration, and redox state [85]. *ENO2*, a membrane protein, has been reported as a marker of *neuro-muscular junctions* (*NMJs*), whose expression decreases considerably during the initial stages of human embryonic muscle tissue development [86]. *VEGF-D* interacts with, and induces dimerization and tyrosine autophosphorylation of its endothelial cell-specific receptor, *VEGFR-2*, which stimulates endothelial sprouting, proliferation, and survival, as well as vascular permeability. Similarly, binding of VEGF-D with VEGFR-3 stimulates related processes in lymphatic endothelial cells [87], [88], [89], [90].

#### Phosphorus metabolic process

**Figure 5:**
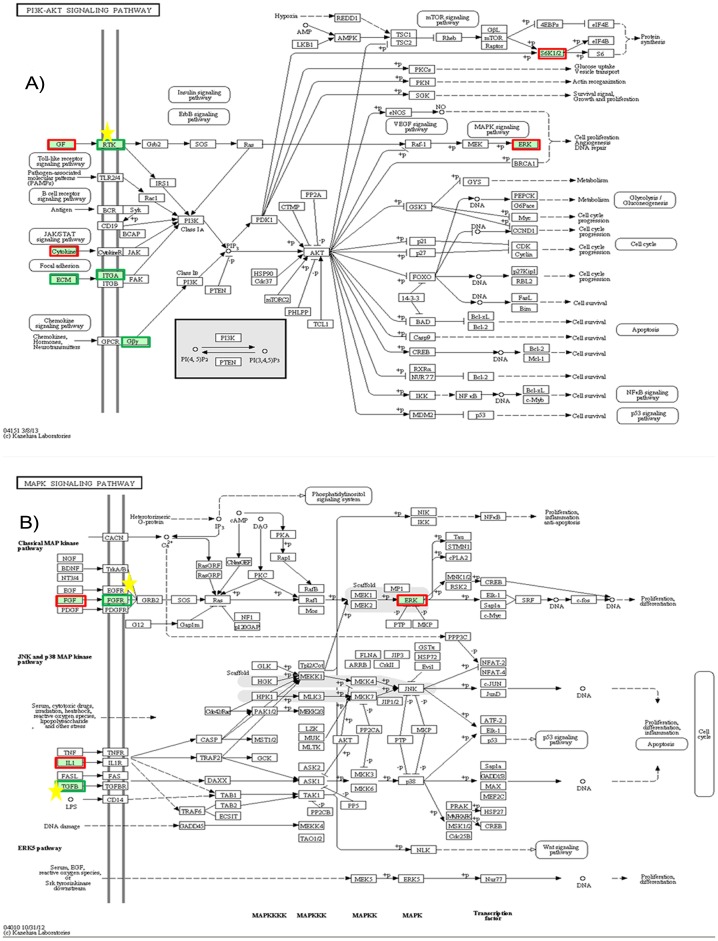
KEGG pathway map analysis of differentially expressed genes (≥4-fold change). Among the various different pathways reported by KEGG analysis, two representative pathways **A**) PI3K-AKT and **B**) MAPK are shown. For each rectangular shape, red and green borders indicate up-regulated and down-regulated genes, while the yellow star indicates a role of phosphoprotein. Each KEGG pathway analysis figure depicts the role or involvement of a specific gene or a family of related genes at a particular location in the pathway. For instance, the FGF gene in KEGG MAPK pathway map represents the other FGF members (such as FGF10, FGF11, FGF12, etc) as well that may or may not be affected. Here, FGF represents all the members of fibroblast growth factor family. For FGF family members, the gene that exhibited >4 fold increase includes FGF11 whereas FGF10, FGF16, and FGF18 showed >1 fold increase in their expression rates. Conversely, the other FGF members that showed <4 fold decreased expression include FGF12 and FGF14. Similarly, FGFR represents the other members (FGFR1, FGFR2, etc) of this family of receptors as well. In case of FGFR family, FGFR2 showed >4 fold decrease in its expression, and FGFR4 exhibited >1 fold decrease in its expression rate. However, FGFR1 and FGFR3 showed about 1 fold increase in their expressions.

FAC analysis identified phosphorous metabolic processes as an important biological process in MyoG_kd_ cells (**Table 3B** **& Table S3B**). Both the FAC and function annotation chart analysis detected about 21 unique genes that exhibited ≥4-fold decrease in their expression rates and were involved in phosphorous related processes. These genes included various phosphatases and kinases such as *receptor-type tyrosine-protein phosphatase beta* (*PTPRB*), *serine/threonine-protein phosphatases PP1-beta catalytic subunit* (*PPP1CB*), *serine/threonine-protein kinase 40* (*STK40*) and *cyclin-dependent kinase 13* (*CDK13*). One of the main mechanisms by which the signaling cascades control various stages of myogenesis is through protein kinases that direct cell behavior via the reversible process of phosphorylation [91]. Extensive studies have revealed that protein tyrosine phosphatases play a vital role in regulation of skeletal muscle myogenesis [59], [92], [93], while dephosphorylation of tyrosine residues is required for cell cycle exit during myogenesis [94]. The KEGG pathway analysis identified various pathways that lead to down-regulation of various protein phosphatases during at least one step of their representative pathway, such as the PI3K-Akt signaling pathway, MAPK signaling pathway, focal adhesion, TGF-beta signaling pathway and hippo signaling pathway (**Figure 5**). One of the down-regulated genes is protein phosphatase 1, catalytic subunit, beta isozyme (*PPP1CB*), which encodes a serine/threonine-protein phosphatase PP1-beta catalytic subunit, an important enzyme responsible for protein phosphorylation and regulation of many physiological processes [95]. Pathway analysis also illustrated that *PPP1CB* is involved in many important pathways related to myogenesis such as focal adhesion, the Hippo signaling pathway and regulation of the actin cytoskeleton (**Figure S3A–E**).

#### Cytoskeleton and cell morphogenesis

DAVID FAC indicated that the GO terms cytoskeleton and cell morphogenesis involved in differentiation were down-regulated in response to MyoG_kd_. The analyses identified about 39 unique genes involved in these processes that showed ≥4-fold reduction in their expression rates. The location of most of the genes in this category is either cytoplasm or labeled as secreted in the Uniprot database. The genes under these biological processes are involved in a broad range of functions such as signaling pathways, transport, differentiation, etc. Some of these genes include *disabled homolog 2* (*DAB2*), *microtubule-associated protein 2* (*MAP2*), *synaptopodin-2 or myopodin* (*SYNPO2*), and *moesin* (*MSN*). Among these genes, *DAB2* (expressed in various tissues), which is detected at an early myogenic differentiation state [67], has lost or reduced expression in hyperproliferative cells [96]. Another gene in this group that is significantly down-regulated is a member of the tissue inhibitors of matrix metalloproteinases (*TIMP*) family, *TIMP3*, or *metalloproteinase inhibitor 3*. *TIMP3* complexes with *MMPs* and is the only *TIMP* capable of inhibiting membrane bound *MMP*, transmembrane *MMP* and sheddases such as TNF-α converting enzyme (TACE), which is also known as disintegrin and metalloproteinase (ADAM-17) [97], [98]. Conversely, all *MMPs* detected in this study were highly up-regulated. Pathway analysis also revealed that one of the ERM proteins known to regulate cross-linking of the plasma membrane and actin cytoskeleton, *MSN*, was down-regulated [99], [100], [101].

### Role of glycogene expression in myogenesis

Skeletal muscle development consists of a well controlled and regulated progression of various cellular processes, including cell proliferation, migration, and differentiation [102]. Until recently, the role of glycoproteins in myogenesis did not receive a great deal of attention from the scientific community [5], [102]. However, many independent studies have recently focused on the numerous roles of glycoconjugates during myogenesis [102]. The results of these studies have indicated that the expression of *MyoG* is partly regulated by the reduced glycosylation-dependent recruitment of *Mef2D* to *MyoG* promoter, suggesting negative regulatory mechanisms of skeletal muscle development by O-GlcNAc glycosylation [103].

Different processes related to the formation and maintenance of skeletal muscles are characterized by the expression of a wide variety of molecules that strongly alter biological events, such as muscle development, differentiation and regeneration. Among the different types of macromolecules participating in myogenesis, interest in glycoproteins has been gaining remarkable attention in recent years; however, there are still several unanswered questions regarding their roles during skeletal muscle development [102]. Similar to other eukaryotic cells, the plasma membrane and ECM of myoblasts are rich in glycoproteins and glycolipids [5]. Inhibition of some ECM proteoglycans (*syndecans*) has shown to stop the progression of myoblast proliferation and fusion, regardless of the expression of MRFs [104], [105]. Similarly, interrupting N-glycan synthesis affects myoblast fusion [106]. Glycolipids also play key roles in cell differentiation and muscle development [107], [108].

### Role of channels in myogenesis

The initial phases of myogenesis are marked by the development of excitability and contractile properties by skeletal muscle cells [109]. Voltage dependent sodium channels comprise one of the key types of proteins that play a pivotal role in propagating action potential in nerves and muscle [110], [111], [112]. *SCN1A* consists of four homologous domains [113], and its activity is regulated by the interaction with *fibroblast growth factor 13* (*FGF13*) [114]. Many mutational studies have recognized a variety of tainted channel properties that include changes in the voltage dependence of activation and inactivation, speedy recuperation from inactivation, improved constant current and loss of channel function [113]. Similarly, *RYR1*, a calcium channel that plays an important role in excitation–contraction coupling in skeletal muscles, has shown increased expression levels during the early stages of myogenesis along with *dihydropyridine receptors* (*DHPRs*) [115]. *SYNC*, which belongs to the *intermediate filament* (*IF*) family of proteins [116], is greatly expressed in skeletal and cardiac muscles and may play an important role in maintaining contractile properties [117]. *SYNC* is known to interact with another member of the IF family, *desmin*, and may play a significant role in protecting muscle cells from mechanical stress [118], [119] in a fashion similar to that of other members of the IF family [120].

Together, these data offer extensive and deeper insight into the transcriptional regulation of myogenesis by *MyoG* and provide a rich scope for designing future experiments to elucidate the pathways involved in skeletal muscle differentiation. Furthermore, to improve muscle growth as well as quality and quantity of meat, it is essential to recognize how *MyoG* influences the differentiation of MSCs.

## Conclusion

In summary, our transcriptome analysis of primary bovine cells using RNA-Seq offers important insight into the transcriptional regulation of gene expression in down-regulated *MyoG* muscle cells. In addition to the identification of new genes in skeletal muscle development, our bioinformatics analysis suggested a role of phosphorous metabolic processes, proteins with channeling function, and the involvement of a significant number of glycogenes in myogenesis. Further investigation of the genes identified in this study will facilitate our understanding and help explain the mechanism responsible for increasing skeletal muscle mass.

## Supporting Information

Figure S1 **Pax7 expression in MSCs.** Cellular localization of Pax7 on MSCs at Day 10 by immunocytochemistry. **A**) Cell picture **B**) DAPI-stained nuclei. **C**) Pax7 antibody stained cells.
(TIF)

Figure S2 **Multiple sequence alignment of LYL1 and other bHLH genes.** Multiple sequence alignment of LYL1 (UNIPROT ID: E1BAR3) protein and other bHLH proteins (MyoD, MyoG, Myf5, and herculin) which are involved in skeletal muscle development. The region in the box indicates high sequence similarity in the bHLH region of these muscle specific proteins.
(TIF)

Figure S3 **Other muscle specific pathways affected by MYOG_kd_.** **A**) WNT signaling pathway, **B**) Focal adhesion, **C**) TGF-beta signaling pathway, **D**) Hippo signaling pathway, E) PPAR signaling pathway.
(PDF)

Table S1 **Primer information.**
(XLSX)

Table S2 **List of up- and down-regulated genes.** Each sheet contains up- and down-regulated genes separately that show at least 4 fold change in their expression.
(XLSX)

Table S3 **GO functional annotation chart.** GO functional annotation chart records for **A**) up-regulated genes, and **B**) Down-regulated genes. Only those terms that have *p*-values ≤0.05 and number of genes in each term ≥5 are shown.
(XLSX)
