# Supplementary figures and images for: Identification of Genes Differentially Expressed in Myogenin Knock-Down Bovine Muscle Satellite Cells during Differentiation through RNA Sequencing Analysis
Source: PLoS One. 2014 Mar 19;9(3):e92447. doi: 10.1371/journal.pone.0092447 (PMC3960249; doi:10.1371/journal.pone.0092447)

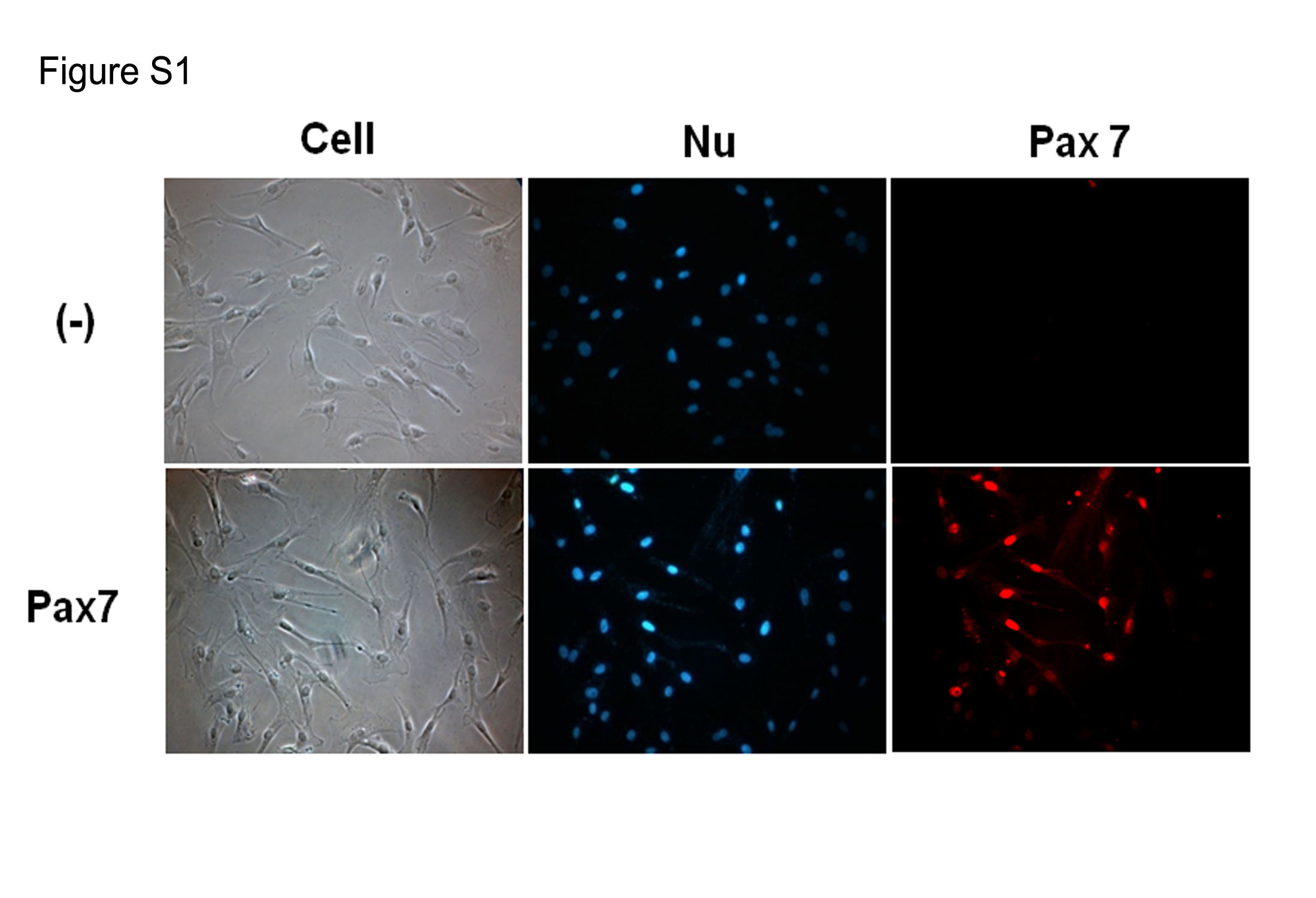

Supplement: Figure S1 — Pax7 expression in MSCs. Cellular localization of Pax7 on MSCs at Day 10 by immunocytochemistry. A) Cell picture B) DAPI-stained nuclei. C) Pax7 antibody stained cells. (TIF) [file pone.0092447.s001.tif]

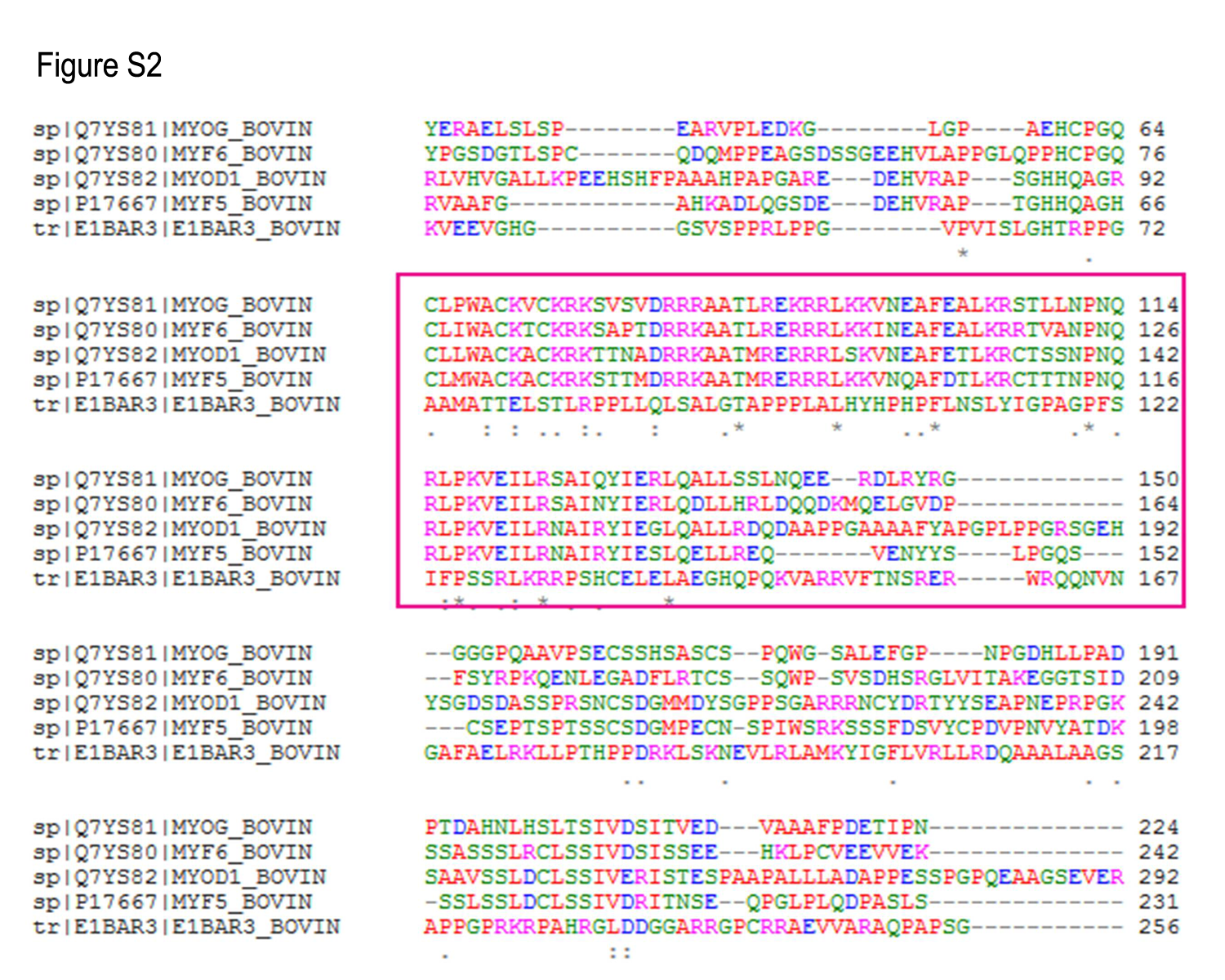

Supplement: Figure S2 — Multiple sequence alignment of LYL1 and other bHLH genes. Multiple sequence alignment of LYL1 (UNIPROT ID: E1BAR3) protein and other bHLH proteins (MyoD, MyoG, Myf5, and herculin) which are involved in skeletal muscle development. The region in the box indicates high sequence similarity in the bHLH region of these muscle specific proteins. (TIF) [file pone.0092447.s002.tif]
