# Supplementary material for: Identification of Genes Differentially Expressed in Myogenin Knock-Down Bovine Muscle Satellite Cells during Differentiation through RNA Sequencing Analysis
Source: PLoS One. 2014 Mar 19;9(3):e92447. doi: 10.1371/journal.pone.0092447 (PMC3960249; doi:10.1371/journal.pone.0092447)
Supplement: Figure S3 — Other muscle specific pathways affected by MYOGkd. A) WNT signaling pathway, B) Focal adhesion, C) TGF-beta signaling pathway, D) Hippo signaling pathway, E) PPAR signaling pathway. (PDF) [file pone.0092447.s003.pdf]

Figure S3A

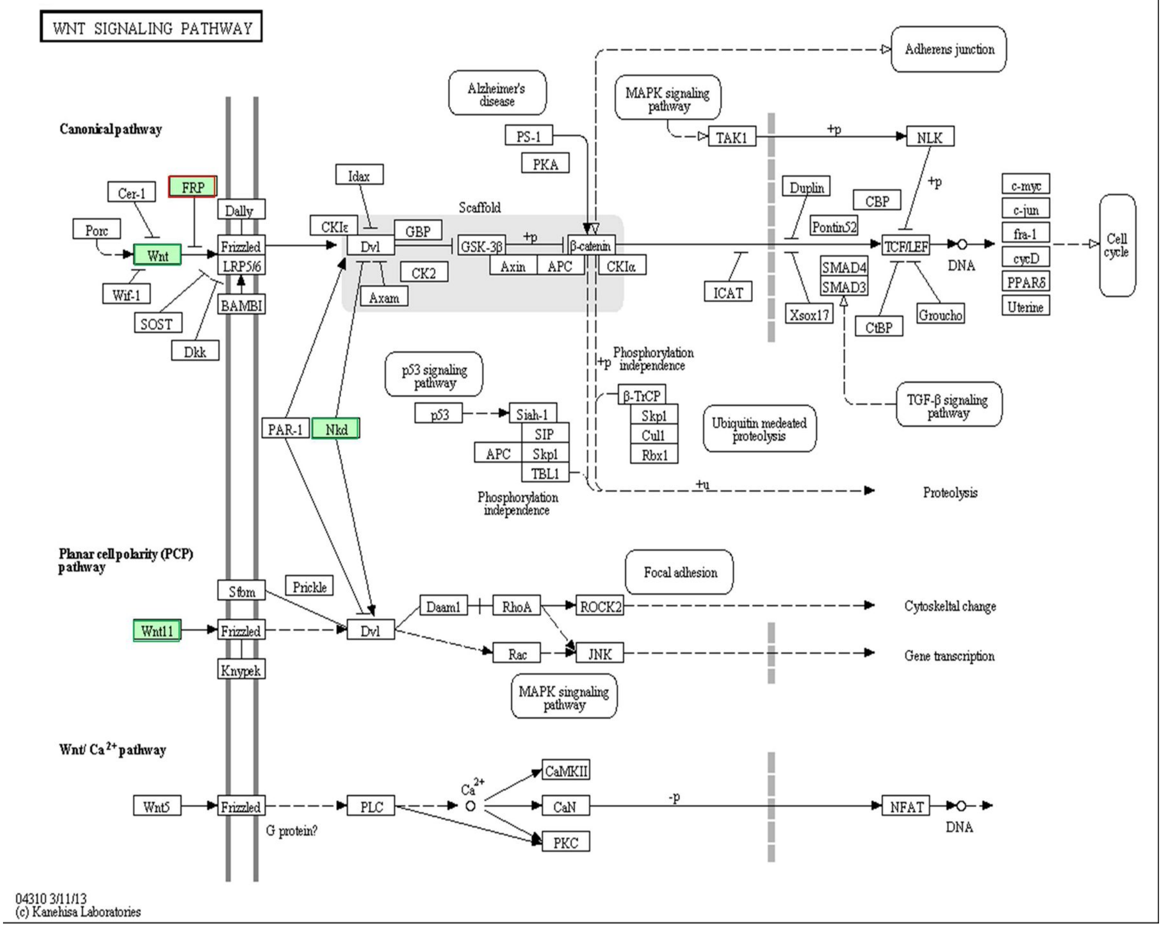
WNT SIGNALING PATHWAY
Canonical pathway
Planar cell polarity (PCP) pathway
Wnt/ Ca2+ pathway
Porc
Cer-1
FRP
Wnt
Wif-1
SOST
Dkk
Dally
Frizzled
LRP5/6
BAMBI
Idax
CKIε
Dvl
GBP
GSK-3β
Axin
APC
β-catenin
CKIα
CK2
Axam
Scaffold
Alzheimer's disease
PS-1
PKA
MAPK signaling pathway
TAK1
NLK
Adherens junction
Duplin
CBP
Pontin52
TCF/LEF
ICAT
SMAD4
SMAD3
Xsox17
CtBP
Groucho
DNA
c-myc
c-jun
fra-1
cycD
PPARδ
Uterine
Cell cycle
TGF-β signaling pathway
p53 signaling pathway
p53
Siah-1
SIP
APC
Skp1
TBL1
Phosphorylation independence
β-TrCP
Skp1
Cul1
Rbx1
Ubiquitin mediated proteolysis
Proteolysis
PAR-1
Nkd
Sfrm
Prickle
Wnt11
Frizzled
Knypek
Dvl
Daam1
RhoA
ROCK2
Rac
JNK
Focal adhesion
MAPK singnaling pathway
Cytoskeltal change
Gene transcription
Wnt5
Frizzled
PLC
G protein?
Ca2+
CaMKII
CaN
PKC
NFAT
DNA
04310 3/11/13
(c) Kanehisa Laboratories

Figure S3B

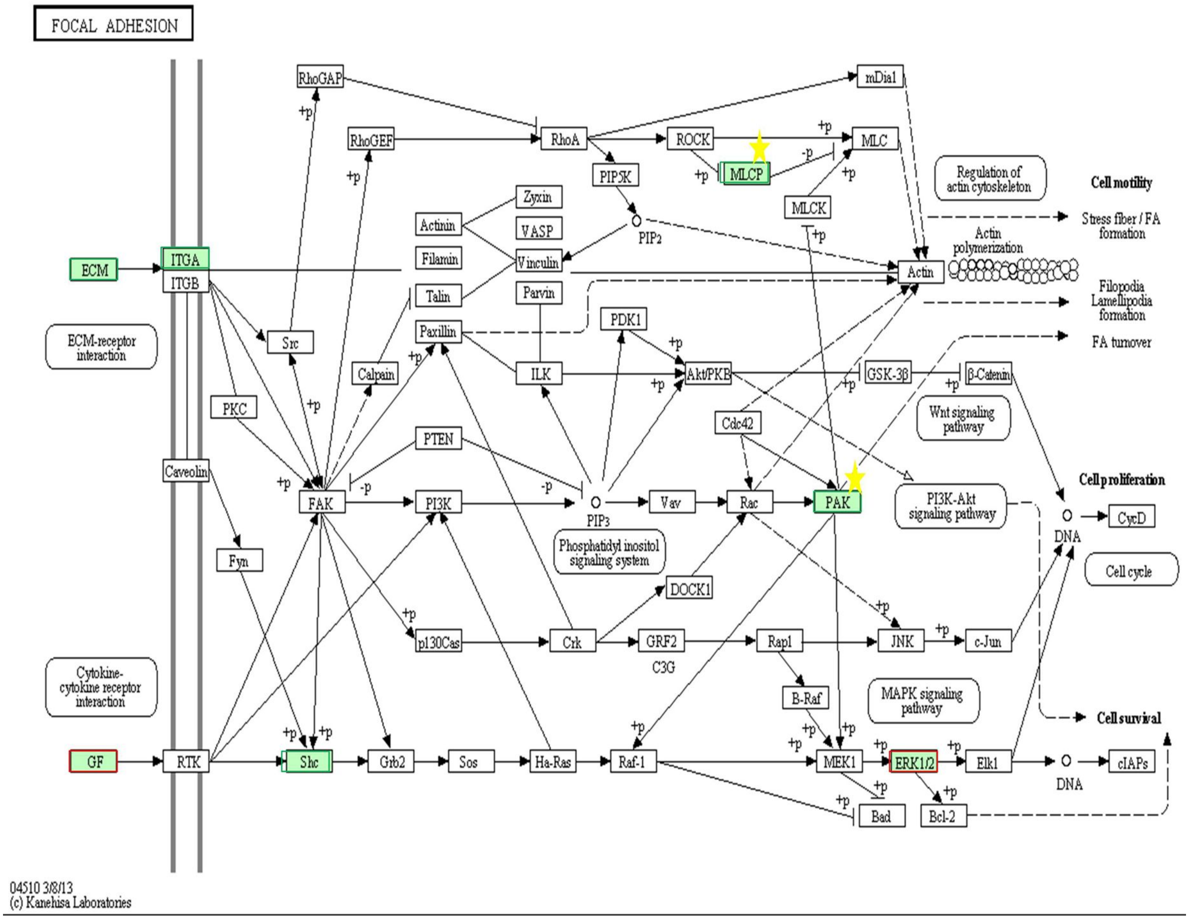
FOCAL ADHESION
RhoGAP
mDia1
RhoGEF
RhoA
ROCK
MLC
PIP5K
MLCP
MLCK
Zyxin
Actinin
VASP
Filamin
Vinculin
Talin
Parvin
Paxillin
PDK1
PIP2
Regulation of actin cytoskeleton
Cell motility
Stress fiber / FA formation
Actin polymerization
Actin
Filopodia Lamellipodia formation
FA turnover
ECM
ITGA
ITGB
ECM-receptor interaction
Src
Calpain
ILK
Akt/PKB
GSK-3β
β-Catenin
Wnt signaling pathway
PKC
PTEN
Cdc42
Caveolin
FAK
PI3K
PIP3
Vav
Rac
PAK
PI3K-Akt signaling pathway
Cell proliferation
DNA
CycD
Cell cycle
Phosphatidyl inositol signaling system
Fyn
DOCK1
p130Cas
Crk
GRF2
C3G
Rap1
JNK
c-Jun
B-Raf
MAPK signaling pathway
Cell survival
Cytokine-cytokine receptor interaction
GF
RTK
Shc
Grb2
Sos
Ha-Ras
Raf-1
MEK1
ERK1/2
Elk1
cIAPs
Bad
Bcl-2
+p
-p
04510 3/8/13
(c) Kanehisa Laboratories

Figure S3C

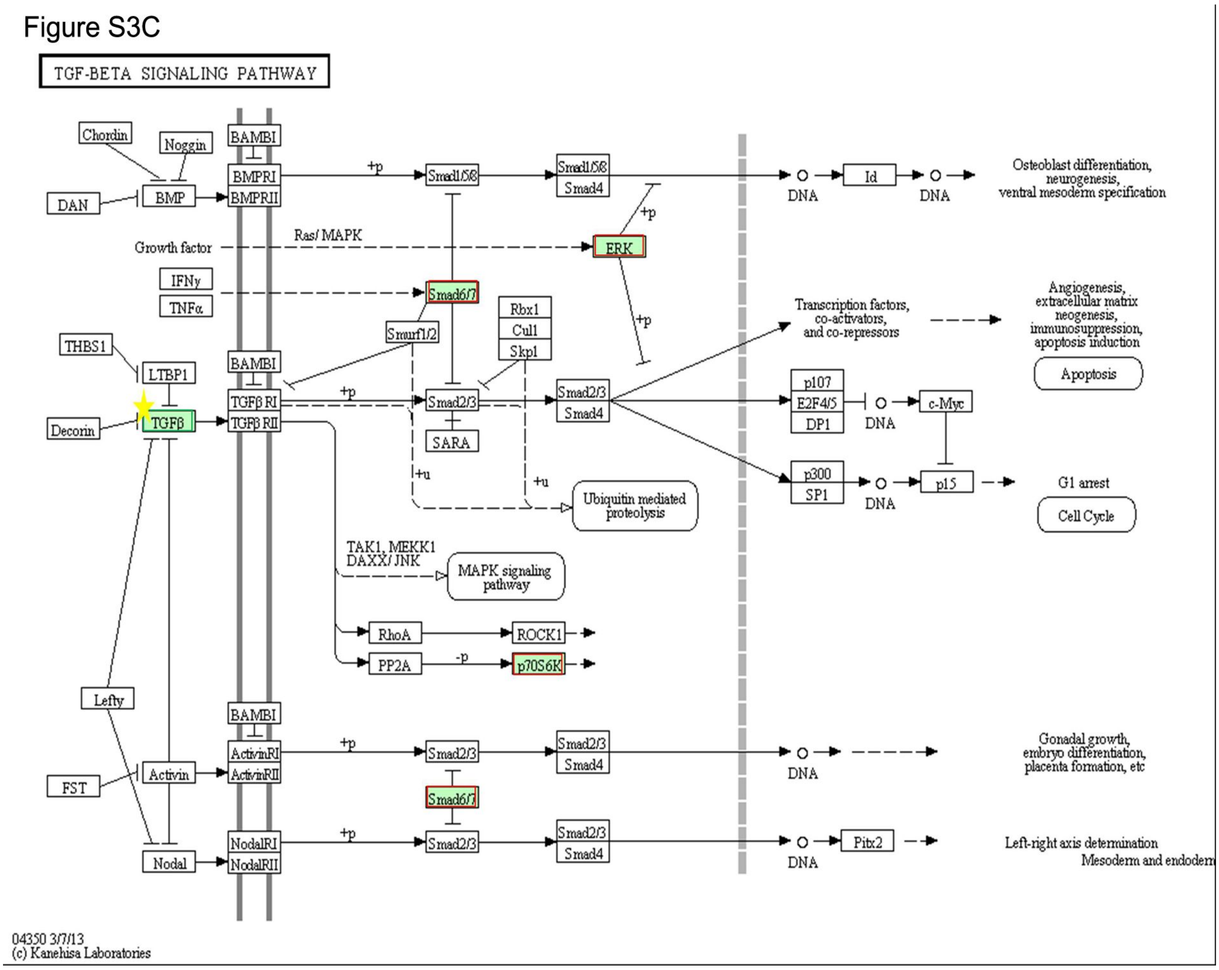
TGF-BETA SIGNALING PATHWAY
Chordin
Noggin
DAN
BMP
BAMBI
BMPRI
BMPRII
+p
Smad1/5/8
Smad1/5/8
Smad4
DNA
Id
DNA
Osteoblast differentiation, neurogenesis, ventral mesoderm specification
Growth factor
Ras/ MAPK
ERK
+p
IFNγ
TNFα
Smad6/7
Rbx1
Cul1
Skp1
Smurf1/2
THBS1
LTBP1
BAMBI
TGFβ RI
TGFβ RII
Decorin
TGFβ
Smad2/3
SARA
Smad2/3
Smad4
Transcription factors, co-activators, and co-repressors
Angiogenesis, extracellular matrix neogenesis, immunosuppression, apoptosis induction
Apoptosis
p107
E2F4/5
DP1
c-Myc
p300
SP1
p15
G1 arrest
Cell Cycle
+u
Ubiquitin mediated proteolysis
TAK1, MEKK1
DAXX/ JNK
MAPK signaling pathway
RhoA
ROCK1
PP2A
-p
p70S6K
Lefty
BAMBI
ActivinRI
ActivinRII
Activin
FST
Gonadal growth, embryo differentiation, placenta formation, etc
NodalRI
NodalRII
Nodal
Pitx2
Left-right axis determination
Mesoderm and endoderm
04350 3/7/13
(c) Kanehisa Laboratories

Figure S3D

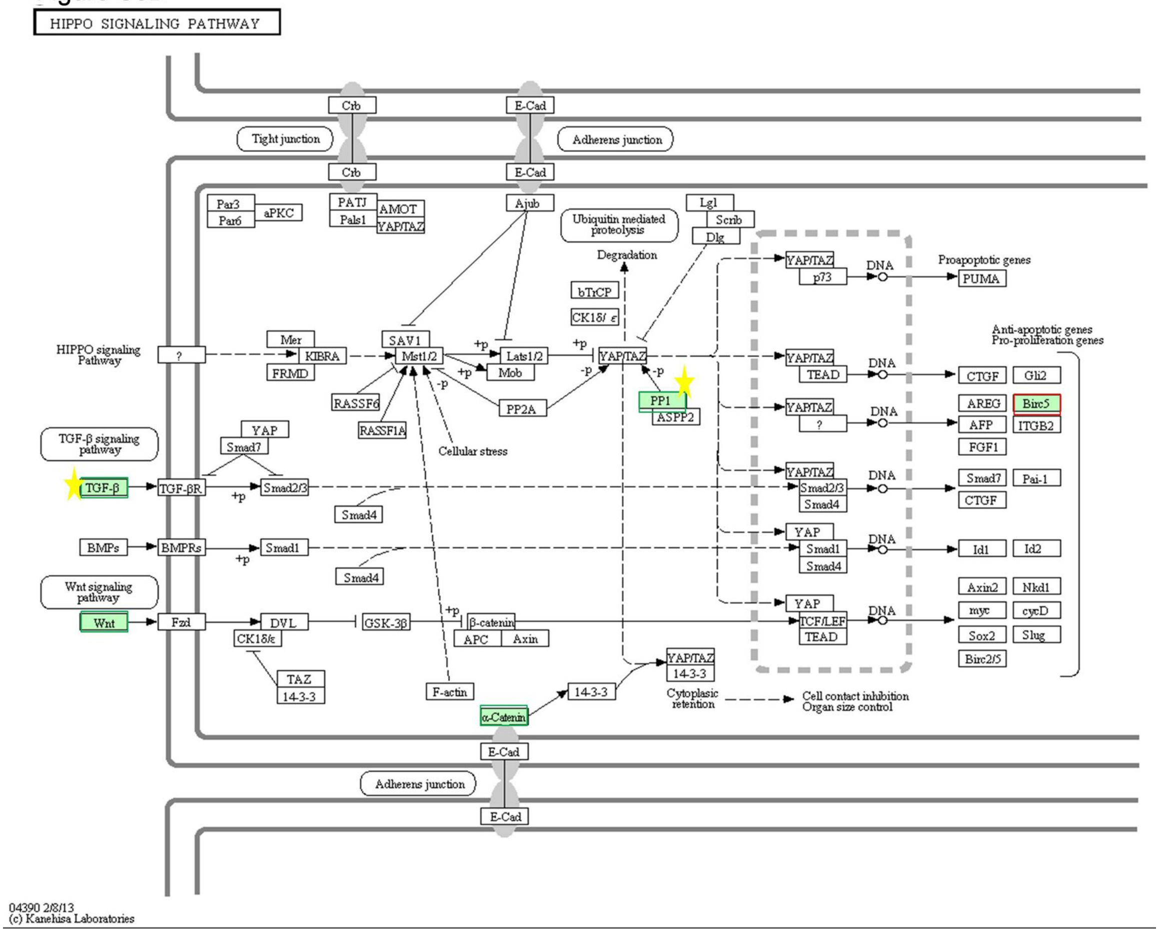
HIPPO SIGNALING PATHWAY
Crb
E-Cad
Tight junction
Adherens junction
Crb
E-Cad
Par3
Par6
aPKC
PATJ
Pals1
AMOT
YAP/TAZ
Ajub
Ubiquitin mediated proteolysis
Lgl
Scrib
Dlg
Degradation
bTrCP
CK1δ/ε
YAP/TAZ
p73
DNA
Proapoptotic genes
PUMA
HIPPO signaling Pathway
?
Mer
KIBRA
FRMD
SAV1
Mst1/2
+p
Lats1/2
Mob
+p
YAP/TAZ
-p
RASSF6
RASSF1A
-p
PP2A
PP1
ASPP2
Anti-apoptotic genes
Pro-proliferation genes
TEAD
CTGF
Gli2
AREG
Birc5
AFP
ITGB2
FGF1
TGF-β signaling pathway
YAP
Smad7
Cellular stress
TGF-β
TGF-βR
+p
Smad2/3
Smad4
Smad2/3
Smad4
Smad7
Pai-1
CTGF
BMPs
BMPRs
+p
Smad1
Smad4
YAP
Smad1
Smad4
Id1
Id2
Wnt signaling pathway
Wnt
Fzd
DVL
CK1δ/ε
GSK-3β
β-catenin
APC
Axin
TCF/LEF
TEAD
Axin2
Nkd1
myc
cycD
Sox2
Slug
Birc2/5
TAZ
14-3-3
F-actin
α-Catenin
14-3-3
YAP/TAZ
14-3-3
Cytoplasic retention
Cell contact inhibition
Organ size control
E-Cad
Adherens junction
E-Cad
04390 2/8/13
(c) Kanehisa Laboratories

Figure S3E

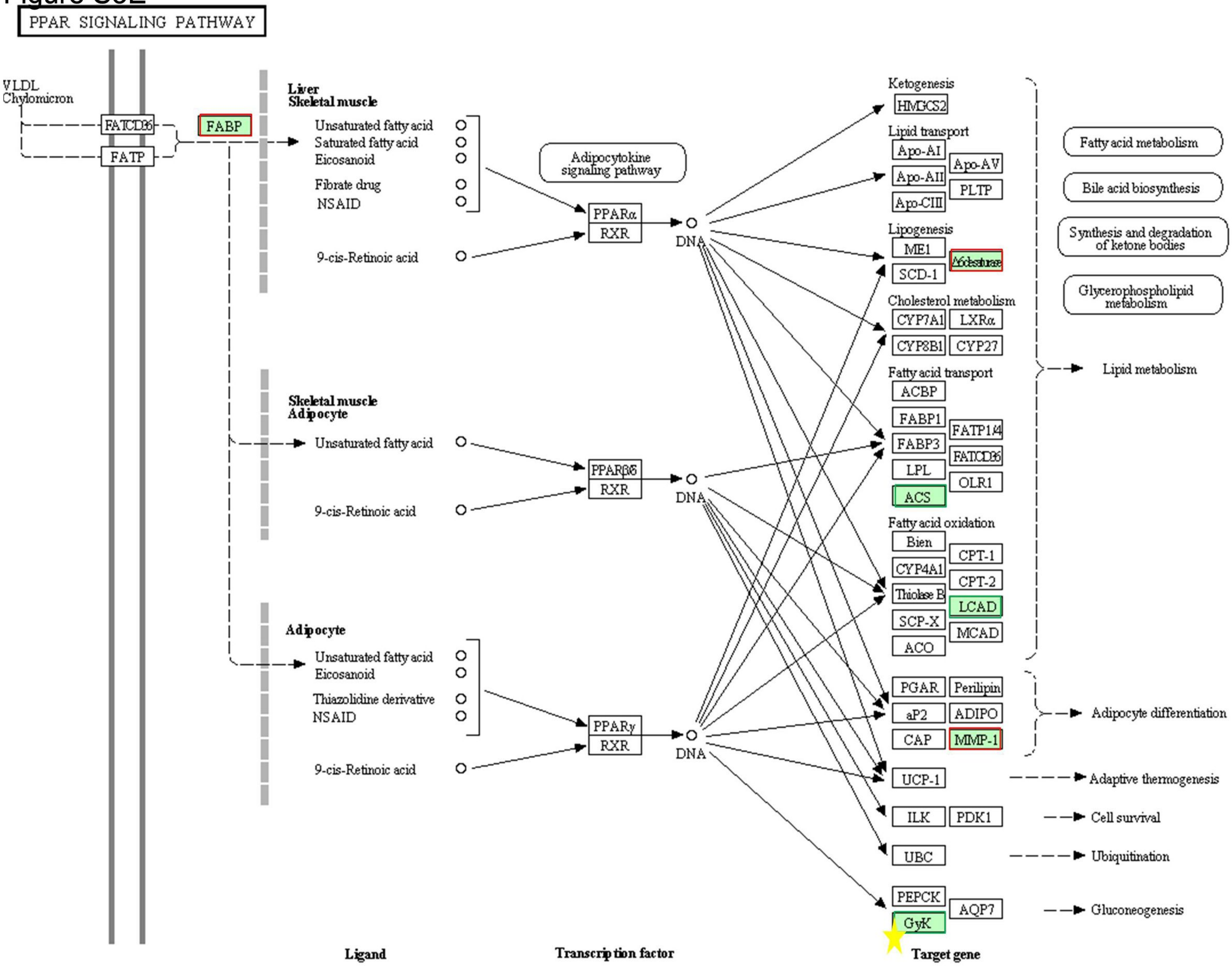
PPAR SIGNALING PATHWAY
VLDL
Chylomicron
FATCD36
FATP
FABP
Liver
Skeletal muscle
Unsaturated fatty acid
Saturated fatty acid
Eicosanoid
Fibrate drug
NSAID
9-cis-Retinoic acid
Adipocytokine signaling pathway
PPARα
RXR
DNA
Skeletal muscle
Adipocyte
Unsaturated fatty acid
9-cis-Retinoic acid
PPARβ/δ
RXR
DNA
Adipocyte
Unsaturated fatty acid
Eicosanoid
Thiazolidine derivative
NSAID
9-cis-Retinoic acid
PPARγ
RXR
DNA
Ketogenesis
HMGCS2
Lipid transport
Apo-AI
Apo-AII
Apo-CIII
Apo-AV
PLTP
Lipogenesis
ME1
SCD-1
Δ6desaturase
Cholesterol metabolism
CYP7A1
LXRα
CYP8B1
CYP27
Fatty acid transport
ACBP
FABP1
FABP3
LPL
ACS
FATP1/4
FATCD36
OLR1
Fatty acid oxidation
Bien
CYP4A1
Thiolase B
SCP-X
ACO
CPT-1
CPT-2
LCAD
MCAD
PGAR
aP2
CAP
Perilipin
ADIPO
MMP-1
UCP-1
ILK
PDK1
UBC
PEPCK
GyK
AQP7
Fatty acid metabolism
Bile acid biosynthesis
Synthesis and degradation of ketone bodies
Glycerophospholipid metabolism
Lipid metabolism
Adipocyte differentiation
Adaptive thermogenesis
Cell survival
Ubiquitination
Gluconeogenesis
Ligand
Transcription factor
Target gene
